# Neural Net Classification Combined With Movement Analysis to Evaluate *Setaria viridis* as a Model System for Time of Day of Anther Appearance

**DOI:** 10.3389/fpls.2018.01585

**Published:** 2018-10-31

**Authors:** Jigar S. Desai, Erin Slabaugh, Donna J. Liebelt, Jacob D. Fredenberg, Benjamin N. Gray, S. V. Krishna Jagadish, Olivia Wilkins, Colleen J. Doherty

**Affiliations:** ^1^Department of Molecular and Structural Biochemistry, North Carolina State University, Raleigh, NC, United States; ^2^Benson Hill Biosystems, Inc., Saint Louis, MO, United States; ^3^Department of Agronomy, Kansas State University, Manhattan, KS, United States; ^4^Department of Plant Science, McGill University, Sainte-Anne-de-Bellevue, QC, Canada

**Keywords:** flower opening time, spikelet opening time, timing of anthesis, setaria, developmental stage, image analysis of plants, biological image classification

## Abstract

In many plant species, the time of day at which flowers open to permit pollination is tightly regulated. Proper time of flower opening, or Time of Day of Anther Appearance (**TAA**), may coordinate flowering opening with pollinator activity or may shift temperature sensitive developmental processes to cooler times of the day. The genetic mechanisms that regulate the timing of this process in cereal crops are unknown. To address this knowledge gap, it is necessary to establish a monocot model system that exhibits variation in TAA. Here, we examine the suitability of *Setaria viridis*, the model for C4 photosynthesis, for such a role. We developed an imaging system to monitor the temporal regulation of growth, flower opening time, and other physiological characteristics in Setaria. This system enabled us to compare Setaria varieties Ames 32254, Ames 32276, and PI 669942 variation in growth and daily flower opening time. We observed that TAA occurs primarily at night in these three Setaria accessions. However, significant variation between the accessions was observed for both the ratio of flowers that open in the day vs. night and the specific time of day where the rate is maximal. Characterizing this physiological variation is a requisite step toward uncovering the molecular mechanisms regulating TAA. Leveraging the regulation of TAA could provide researchers with a genetic tool to improve crop productivity in new environments.

## Introduction

Most crops have been bred for maximum productivity in specific geographical locations and under particular management practices. In a time when weather patterns are changing the ranges in which many staple crops are grown, management practices such as planting dates and watering regimes that were optimized for one region, must be re-optimized for growth at new latitudes (Walthall et al., 2013; Challinor et al., 2014). One mechanism by which plants coordinate their activity with the local environment is through the temporal control of developmental transitions (Harmer, 2010; Bendix et al., 2015; Greenham and McClung, 2015; Nakamichi, 2015). The coordinated timing between developmental stages and environmental conditions is controlled by the endogenous circadian clock and provides an adaptive advantage (Dodd, 2005; Yerushalmi and Green, 2009; Shrestha et al., 2014; Brambilla et al., 2017). The regulation of the developmental transition to flowering in response to photoperiod has been well characterized in Arabidopsis and has provided targets for agricultural selection and biotechnological advances (Blümel et al., 2015; Greenham and McClung, 2015; Hill and Li, 2016; Brambilla et al., 2017; Okada et al., 2017). The appropriate timing of many other developmental transitions is important for optimal coordination with the local environment, yet little is known about the molecular control of these transitions, particularly in monocot species.

The time of day of anther appearance (**TAA**), or the time of day at which flowers open in preparation for pollination, is a developmentally regulated process in dicots and monocots. Also known as time of day of flower opening time, it has been best studied in dicot species, where TAA often must coincide with pollinator activity (van Doorn and Kamdee, 2014). Many monocots, however, are wind or self-pollinated, and so TAA is constrained by other factors. TAA may be genetically and/or environmentally controlled to coincide or avoid particular environmental conditions (De Vries, 1974; Ichimura and Suto, 1998; Kobayasi et al., 2010; Colquhoun et al., 2011; Julia and Dingkuhn, 2012; Lukac et al., 2012; van Doorn and Kamdee, 2014; Aiqing et al., 2018). For example, heat-sensitive reproductive events, including anther dehiscence, pollination, and fertilization could be damaged by exposure to the hottest parts of the day resulting in spikelet sterility and corresponding decrease in agricultural yield (Jagadish et al., 2007; Kobayasi et al., 2010; Bita and Gerats, 2013; Prasad et al., 2017). Unlike the well-studied transitions between vegetative and reproductive phases of development, little is known about the molecular control of TAA in any species; some molecular controls have been identified in dicots (van Doorn and van Meeteren, 2003; Colquhoun et al., 2011; van Doorn and Kamdee, 2014), but much less is known in monocots.

The majority of cultivated rice varieties show little variation in TAA, which almost exclusively occurs within 5 h after dawn (Nishiyama and Blanco, 1981; Prasad et al., 2006; Sheehy et al., 2007; Cattivelli et al., 2008; Bheemanahalli et al., 2017). In contrast, wild rice varieties tend to show wide variation of TAA, ranging from early morning until after dusk. An early morning flowering (**EMF**) introgression line was identified (EMF20) from a cross between a wild accession of rice (*O. officinalis*) and a highly productive cultivated variety (*O. sativa, IR64*). In the EMF20 introgression line, the TAA was advanced by 1.5 h. Because the flowers opened at an earlier and cooler time of the day, the effect of high temperature on spikelet sterility during anthesis was greatly reduced in the greenhouse (Ishimaru et al., 2010) and field conditions (Bheemanahalli et al., 2017). Additionally, two EMF near isogenic lines (NILs) were generated in the Nanjing 11 and IR64 background. Both EMF NILs retained the ability to advance TAA and this earlier flowering resulted in significantly lower levels of spikelet sterility when exposed to heat stress during the day, demonstrating the effectiveness of the trait in both temperate and tropical indica backgrounds (Ishimaru et al., 2012; Hirabayashi et al., 2015; Bheemanahalli et al., 2017). A second EMF introgression line was also developed by crossing *O. rufipogon* and *O. sativa* cv Nipponbare, advancing TAA by about 3 h indicating that this control of TAA can extend to japonica cultivars of rice as well (Thanh et al., 2010). Although genetic control of TAA in rice has been confirmed by these studies, the molecular mechanisms have not been identified. Nor has this been expanded to determine if TAA is genetically controlled in other cereal species.

There are only a handful of TAA studies in other cereal species. In spring wheat, TAA occurs most often either in the early morning or the late evening coinciding with cooler atmospheric temperatures (de Vries, 1972; Aiqing et al., 2018). Unlike in rice, TAA in wheat appears to be strongly affected by environmental conditions; for example, high temperature stress during the day shifts TAA to later evening periods, resulting in flowers opening after the heat stress was released (Aiqing et al., 2018). Variability in TAA and the duration of flowering has been observed for many different wheat accessions (de Vries, 1972; De Vries, 1974). In a study of two northern European wheat varieties, the majority of TAA took place at midday within a span of 2–3 h (Lukac et al., 2012). Lukac et al. hypothesized that a wider range of TAA may improve heat resilience of wheat spikelets compared to varieties that exhibit narrow TAA characteristics.

The narrow range of TAA in domesticated rice cultivars and observed variability in wheat raises the question of whether TAA may have been a selected trait during domestication of some grasses. Identifying the mechanisms that control TAA will offer strategies to adjust TAA to regional environments. Yet, evaluation in controlled conditions will be necessary to fully distinguish between environmental and genetic influences on TAA. *Setaria viridis*, an emerging model for C4 grasses (Brutnell et al., 2010, 2015; Li and Brutnell, 2011; Bennetzen et al., 2012), may provide the opportunity to interrogate TAA in a rapidly growing, smaller stature, transformable plant system with increasing genetic resources. Three studies report observations on TAA in *Setaria* species. Siles et al. (2001) reported that for seven *Setaria italica* cultivars, TAA spanned most of the day for cultivars with elliptical seeds, and was restricted to between just before dawn and just after dusk for cultivars with round seeds (Siles et al., 2001). In a study that developed a crossing method for the common lab variety of *S viridis*, A10, Jiang et al. showed that TAA was primarily restricted to 1 h after dawn (Jiang et al., 2013). Another study analyzed TAA in the A10 variety, imaging of panicles at 1 min intervals over the course of 3 days and concluded that the majority of TAA in A10 takes place at night (Rizal et al., 2013). However, light and touch may induce flower opening (Kobayasi, 2012). In these previous studies fluorescent lights were used to take the night images or the number of open flowers was determined manually which may have interfered with the natural pattern of TAA.

To evaluate the feasibility of Setaria as a model for TAA we established a means for automating TAA analysis, so that the measurement itself does not influence the rate of flower opening. We developed an imaging system and processing pipeline that can track and classify flower opening events in both day and night conditions. We applied our pipeline to three Setaria accessions grown in identical environmental conditions and determined that there are significant variations in TAA between the accessions. Using our imaging conditions, we also evaluated other temporal traits in these accessions and determined that leaf emergence also shows time of day variation between accessions.

## Materials and methods

### Plant materials and growth conditions

Ames 32254, Ames 32276, and A10 (PI 669942) *S. viridis* seeds were obtained from GRIN (www.ars-grin.gov). A10 is a commonly used laboratory variety (Brutnell et al., 2010; Bennetzen et al., 2012). Although originating from Manitoba, Canada, A10 has been propagated in laboratory conditions for several generations. Ames 32254 and Ames 32276 originate from Manitoba, Canada and Bordeaux, France, respectively (Supplementary Figure 1). To break seed dormancy, 10–20 seeds from each accession were incubated with 1 mL concentrated sulfuric acid (Fisher, Hampton, NH) for 10 min, with gentle agitation. Seeds were rinsed four times with 1 mL sterile water. Sulfuric acid did not break dormancy in A10 seeds, therefore these were incubated in 1 mL 0.1% KNO_3_ (Fisher, Hampton, NH) overnight at room temperature. The following day, the seeds were rinsed three times with water, then treated the same as the other varieties. Treated seeds were then placed in wet paper towels in a clear plastic container covered with plastic wrap to maintain moisture, and then placed in a growth chamber for 5–7 days. Seedlings were transplanted to soil that was composed of three parts MetroMix360 and 1-part vermiculite. Plants were fertilized three times a week with Peter's solution (NPK: 20-20-20). Chamber conditions were set to 12 h light: 12 h dark, under 4500–6500 K fluorescent lights, with a daytime temperature of 28°C and nighttime temperature of 23°C. During imaging, plants were grown in the presence of IR light at night (880 nm). Every 3 weeks 10–20 seeds were imbibed for these three Setaria accessions. When the plants transitioned to flowering the panicles were imaged using the platform described below.

### Raspberry pi imaging platform

Raspberry Pi single-board computers (https://www.raspberrypi.org/) were affixed with a NoIR Camera Board for infrared (IR) imaging capabilities. An IR light source was built according to the protocol developed by the Maker Group at the Danforth Center (http://maker.danforthcenter.org/). The camera and IR light source were mounted on ring stands. The focal length of the camera was manually adjusted to 10.2 cm. One image was taken every minute and images were immediately uploaded to a server using the sshpass function. Panicles were imaged for 3–4 days until flowering was complete. The image acquisition parameters for nighttime images are sensitive to the distance of IR source to object and the IR signature of the object. The optimal image acquisition parameters for night images were determined using the raspistill function and a custom optimization loop (https://github.com/DohertyLab/Setaria-Flower-Opening-Time). This loop tested all combinations of each camera option. The best camera option combination was scored by number of edges detected in night images. “–drc high –br 45” were added to night image options based on optimization scores. A sample dataset for the A10 images in both the day and night has been uploaded to https://dataverse.harvard.edu/dataverse/doherty.

### Quantification of open and closed flowers

Flower opening time was determined by two classification methods depending on light source. In the day the visible light spectrum provides (RGB) features to help classification of open and closed spikelets. In these conditions, neural network classification approaches excel at image classification and successfully classify open flowers for images taken in light. To apply these approaches, individual spikelets were monitored. Day time panicle images with a variety of spikelet flowering stages were used to subset spikelets into images of closed spikelets, open spikelets, and background images using LabelImg (https://github.com/tzutalin/labelImg, version 1.4.3). LabelImg was used to create an xml file of coordinates within images that indicate a location with either closed spikelets, open spikelets, or background points. An xml file was created for every panicle image used in the training set. Using the xml file and the original panicle image, all labeled xml coordinates were cropped out and converted to a list of small cropped images of closed spikelets, open spikelets, and background. This was accomplished using R package raster (version 2.58) to upload and store images in R and R package XML (version 3.98) to read the xml file into R. The images were then scaled to 34 × 34 images to meet the dimension input of Inception V3 neural network structure, the classifier we used (Szegedy et al., 2016). The list of images was converted to a 4-dimensional array. Where the 3rd dimension held RGB values and the 4th dimension held the picture order, which indicates the order of the images in the training set as they were used for learning. To summarize the input training set array had a structure of [1:34,1:34,1:3,1:6500], 1:34 is the image dimensions, 1:3 are the RBG values, and 1:6500 is the order of the images. Of the 6,500 training images 5,000 were background images, 1,000 were closed spikelets, and 500 were open spikelets. A separate vector contained label information (closed, open, and background) of the 6,500 training images. A validation set was created containing 600 images which included 500 background images, 50 closed spikelets, and 50 open spikelets. For training, mxnet's mx.model.FeedFoward.create was used to create a classification model. For the neural network configuration Inception V3 34 × 34 was used, for train.x input the described array from above was used, and for train.y the vector containing image labels was used. Other modified parameters include: num.rounds = 250, learning.rate = 0.0001, and momentum = 0.9). The validation set was scored with the eval.data option. After 250 rounds, the model reached 100% classification accuracy on the training set and 99.5% accuracy on the validation set, and 98% accuracy on the open vs. closed spikelet images (Supplementary Figure 2). The training phase took approximately 8 h per fifty iterations and the 250 rounds took 42 hours.

The saved model was loaded using mx.model.load. Each daytime image was segmented into 34 × 34 images as described above. If an image's dimension was not divisible by 34, it was then resized using the raster package to the closest dimension that would evenly divided by 34. For example, a 1000 × 500 panicle image would be resized to 986 × 476. Using the base R function, predict, classification probabilities of each image as closed, open, or background was determined using the same structure as the training set. The classification with the highest probability is used for the image call. To reduce processing time per image, background area was cropped from the panicle image. Raster select was used on an image stack to crop each image to the panicle region. A function was created to integrate this workflow for day time image classification. Both the function and the model can be found at https://github.com/DohertyLab/Setaria-Flower-Opening-Time.

### Imaging flower opening time in the dark

All images from the 12 h night period were extracted and combined with the last image taken in the light. The last light image was used to identify the location of every spikelet as described above. The 34 × 34 images were then run through the day time flower neural network and used to identify open and closed spikelets. For the spikelets identified as closed, each spikelet image stack was cropped out to create a time series of images per spikelet (Supplementary Videos 1–3). Each spikelet imaged was analyzed independently and segmented into 30-min sections. The first image in each 30-min section was then subtracted from each image in the 30-min series. If at least 5% of the pixels in a spikelet image showed a change in pixel intensity that was greater than 50% of the max pixel intensity the time point of that image was considered the TAA. Often the movement associated with flower opening spanned several minutes, therefore, if a spikelet had more than one time segment with enough movement to be classified as opening, the change at the earliest time point was considered the TAA. To adjust for movement due to random vibrations or growth of the panicle the whole image was adjusted by change in the center of pixel intensity. Each consecutive image was adjusted by the previous center of pixel intensity at the previous time point so that the center of intensity was equal in all images. Validation of TAA calls for night images were confirmed by comparison to flower opening calls in the light period, that is a flower cannot open both at night and in the day, and by human assessment.

The rate of TAA was determined by calculating the total number of open flowers in a given time segment over the number of spikelets identified. For night images, the flowering time for all spikelets is combined and cumulatively summed. The cumulatively summed flowering time represents the increasing number of open flowers over the night period. This vector was then scaled to the ratio of open and closed spikelets of the previous day image and the first day image of the next day. This ratio is plotted over several day and night periods (Supplementary Figure 3). To find the rate of flower opening, R's smooth.spline was used to fit a function to the ratio of open and closed spikelets over time. R's deriv function was then used to find the rate of flower over time.

The *post hoc* test of the variance of the rate of TAA between accessions in the 1.5 h after dusk was performed using pairwise.t.test from the R stats package using Holm's adjustment for controlling Type I error.

### Bristle density classifier

Bristle density was calculated by dividing the number of bristles by the total height of the panicle in the image. Bristles were identified by classifying the base pixels of the bristles and the tip pixel of the bristle. If the base of a bristle was able to be connected to a tip by continuous white pixels, then the bristle was counted. The panicle dimensions were calculated from the classified bases of each bristle. Regression lines were plotted through the bases and used to estimate the panicle dimensions. The total height of the panicle was determined by using the bottom of the image and the top intersection point of the regression lines. If the regression lines never intersect, then the row of the first white pixel not classified as a bristle from the top of the image was used. The bristle density was then calculated for each image in a set. All day time images after panicle emergence were averaged to determine the final bristle density for that panicle. Scripts are available at https://github.com/DohertyLab/Setaria-Flower-Opening-Time.

### Quantification of growth

ImageJ Fiji (https://imagej.net/Fiji/Downloads) was used to analyze the daily growth pattern of seedlings from 3 to 10 days after emergence. Dawn and dusk images at 8 a.m. and 8 p.m. were imported as an image sequence. In three replicates of each variety the second and/or third leaves were isolated first through manual cropping using the freehand selection tool and edit->clear function followed by applying a color threshold. The color threshold was adjusted and applied to the stacked images with the following parameters: hue range 39–113, saturation range 30–187, brightness range 0–255, thresholding method default, threshold color B&W, and color space HSB. Image color was then inverted and a script was applied to calculate the midpoint and length along the midpoint.

The setaria_growth_midpoint script inputs the jpeg image files, calculates the midpoint at all points along the leaf, then determines the length of the leaf from this midpoint. The total length is reported in a csv file. The leaf image is oriented vertically and the script iterates over the image, pairing white pixels across the width of the image for each row containing pixels. The pair of pixels of interest was then selected to calculate the midpoint. For all image analysis here, the leaf was oriented to be vertical in the image and we selected the left- and right-most white pixels in each row to calculate the midpoint. However, the provided script allows the user to specify this choice to adapt to different images. After the midpoints are determined along the length of the leaf, the length of this midline is calculated using the distance formula. If the line of midpoints is broken, then the distance is calculated from the two closest midpoints determined.

### Quantification of time of leaf emergence

The time and date of each emergence event was recorded manually using hourly images of 3- to 22-day-old seedlings. The number of emergence events at each hour was calculated using the “COUNTIF” excel function. Varieties were scaled using the number of emergence events for any given hour divided by total emergence events. The data was then graphed using the smooth.spline function in R with a degree of freedom of 3 as confirmed by a cross validation.

## Results

### Determining TAA during light periods

**Figure 1:**
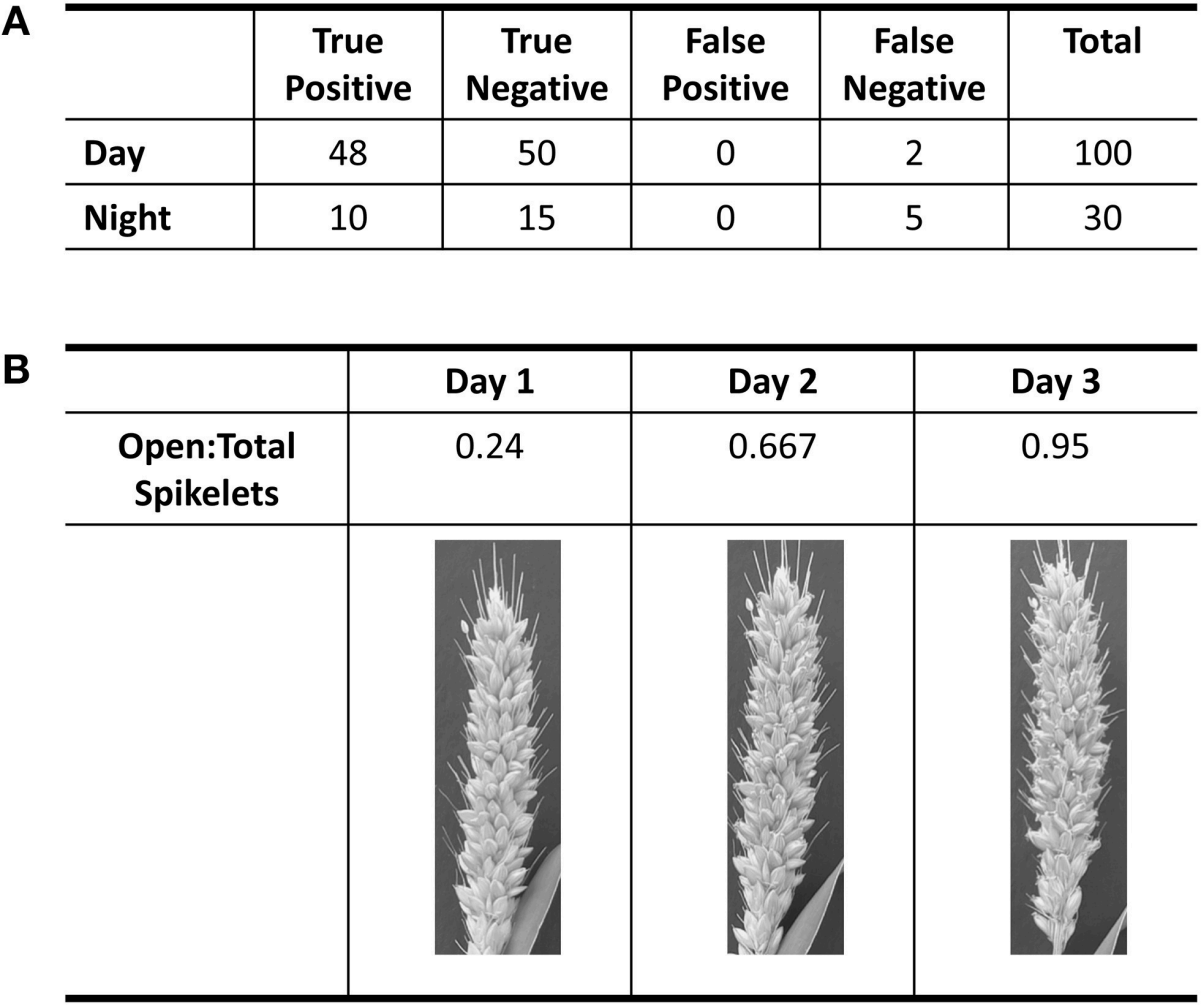
Evaluation of accuracy in flower opening analysis in A10 accession against human classification of images. **(A)** Table of results from the validation of day and night classification models. Validation of daytime classification was tested against 600 hold-out images. Validation of flower opening calls in IR images (Night) was tested against 30 time-lapse videos. True positive and true negative are flowers that were correctly called as “open” or “closed,” respectively. **(B)** Table of the progression of the ratio of open spikelets to total spikelets for three daytime images based on the classification model for the daytime.

Using the A10 accession of Setaria, we optimized our image capture and analysis platform. Images were taken every minute after panicle emergence until plants showed no more flower opening for a 24 h period using a Raspberry PI camera system. An IR light source (http://maker.danforthcenter.org/) was constructed and used for nighttime imaging. For each spikelet, the TAA for daytime images was quantified using Inception V3 convolutional neural network structure (**InceptionV3cnn**) (Szegedy et al., 2016). The training set consisted of 5,000 background images (e.g., leaf, stem, bristles, or other non-panicle images), 1,000 closed spikelets, and 500 open spikelets classified by two researchers. The model was trained until the prediction accuracy for the holdout validation set of 600 images (500 background, 50 closed spikelets, and 50 open spikelets) exceeded 99% (Figure 1A). Images of the A10 accession taken at 10-min intervals were then evaluated using this trained classifier. Each spikelet was classified as background, closed spikelet, or open spikelet. Each analysis was evaluated to ensure that the percent of open panicles increased with time (Figure 1B) and the initiation of flower opening and full flower opening agreed with human assessment as verified independently by two individuals.

### Determining TAA in night images

**Figure 2:**
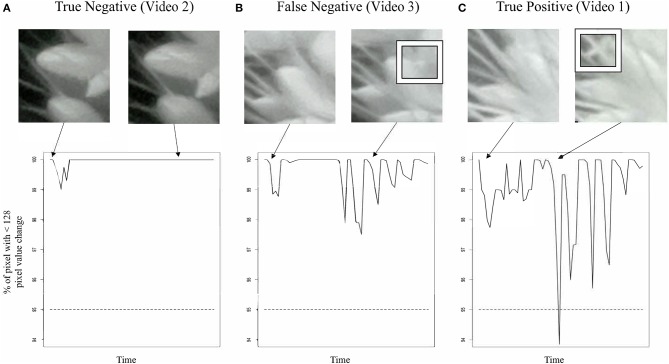
Classification of TAA in nighttime images. Snapshots from nighttime image set (top row) and graph of movement detection (bottom row) for three selected spikelet nighttime image sets. **(A)** A true negative example. A spikelet classified as not opening that is validated as not opening in this 12 h time series, corresponds to Video 2. **(B)** A false negative example, a spikelet classified as not opening that does open. Inset box highlights anther appearance, corresponds to Video 3. **(C)** A true positive example, a spikelet classified as opening that does open in the 12 h time series. Inset box highlights anther appearance, corresponds to Video 1.

We were unable to train the InceptionV3cnn classifier to correctly distinguish between open and closed flowers in the nighttime images. Images taken of plants illuminated by the IR light lacked the visible spectrum features the classifier used to distinguish between open and closed flowers. Therefore, we developed an approach to detect flower opening in nighttime images based on the movement of the flowers as they open (Figure 2, Supplementary Videos 1–6). A time series image stack was made for each spikelet that remained unopened in the last image of the day. The movement of each spikelet was monitored in 30-min intervals throughout the night images. A spikelet was determined to be flowering in the 30-min window if 5% of pixels showed a change in intensity greater than 50% of the total intensity value in the 30-min window. To account for background movement of the spikelet, the center of pixel intensity was used to adjust the frame around the whole panicle image. That is, as the center of pixel intensity for a spikelet moved relative to the frame, the entire frame was adjusted with the same change in intensity. The 30-min window with the earliest movement was classified as the TAA for that spikelet. The correct classification of TAA at night was validated by manually viewing 30 spikelets time series and identifying spikelets that were correctly called as transitioning to an open flower. Accuracy of nighttime flower opening was 83.3% (Figure 1A). Error in flower opening classification exclusively came from false negative calls (Figure 1A). Lowering the sensitivity for flower opening introduced more false positives. As our objective was to identify the rate of flower opening, we elected to proceed with the approach that used the higher sensitivity and calculate TAA as the rate of the detected flowers that open. The established night time flowering protocol was efficiently detecting flower opening time and could address variability in TAA across different accessions. Once the imaging setup and analysis pipeline was successfully established for the A10 accession, we then assessed its performance on other accessions.

### Variation in bristle density impacts daytime classification of flower opening

**Figure 3:**
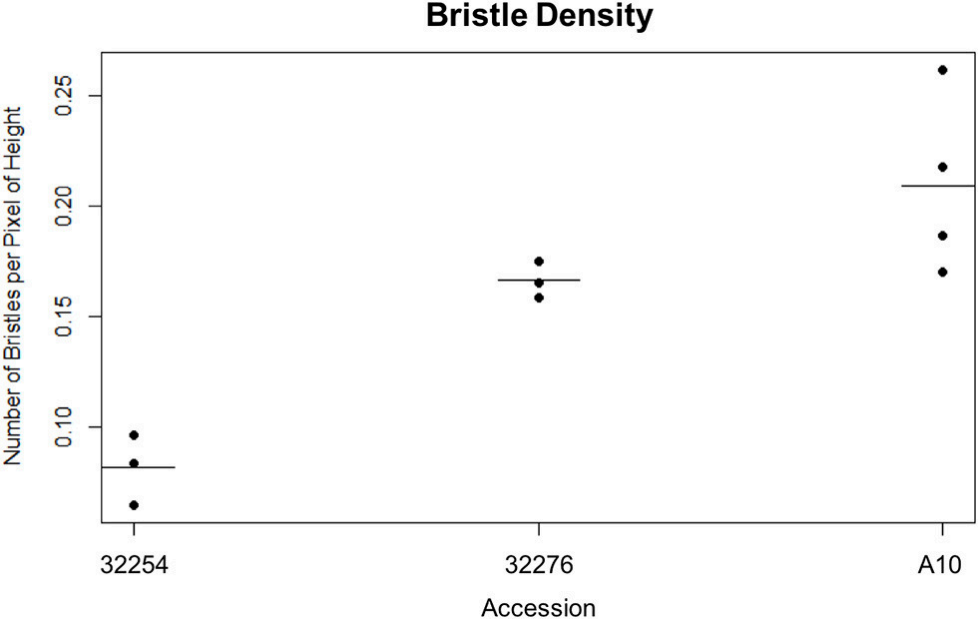
Dot plot of the bristle density of A10, Ames 32254, and Ames 32276 (Student's *t*-test * < 0.05, *N* = 3) bar indicates mean for each accession.

We imaged panicles for three accessions of *Setaria viridis:* Ames 32254, Ames 32276, and A10. We selected these lines because they consistently produced panicles in our chamber conditions and spanned a range of latitudes (49.64306000, 44.82616900). We tested both our day and nighttime TAA classification pipeline on these three accessions and found that while our nighttime movement algorithm performed equally well for all accessions, the variation in bristle density affected the performance of the daytime image analysis (Figure 3). Our early versions of the InceptionV3cnn reached peak performance only when trained with a data set of similar bristle density. Therefore, in anticipation of evaluating multiple accessions, we developed a classifier to categorize bristle density from a given training set (Figure 3). However, when properly trained with panicle images from each of the three accessions the InceptionV3cnn classifier performed equally well on all three accessions, and only one model was required.

### Setaria accessions show variation in TAA

**Figure 4:**
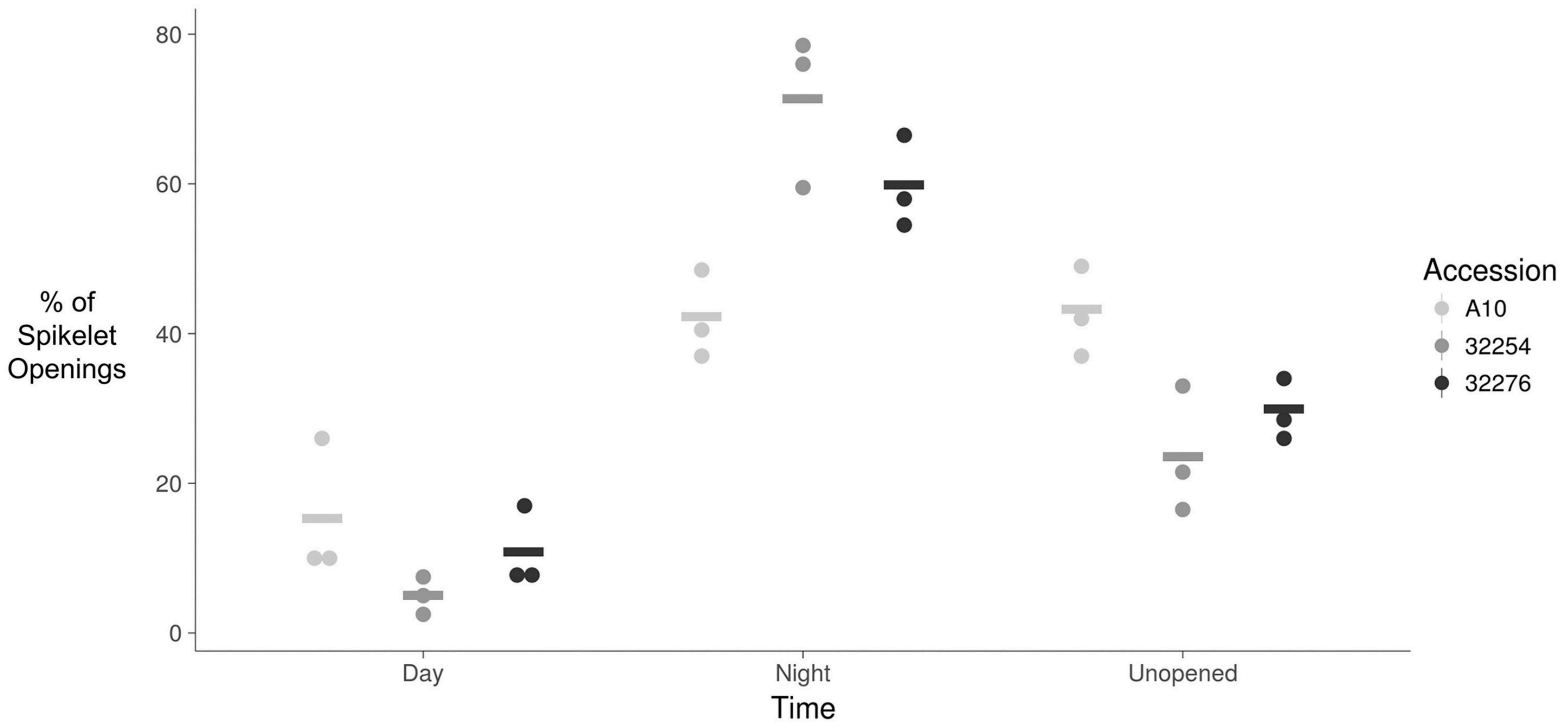
Dot plot of percent of flower opening that occurs during the day, night, or remains unopened for A10, Ames 32254, and Ames 32276. Data represents all flowering opening events that were captured over a 3 day period (*N* = 3) bar indicates mean for each accession.

**Figure 5:**
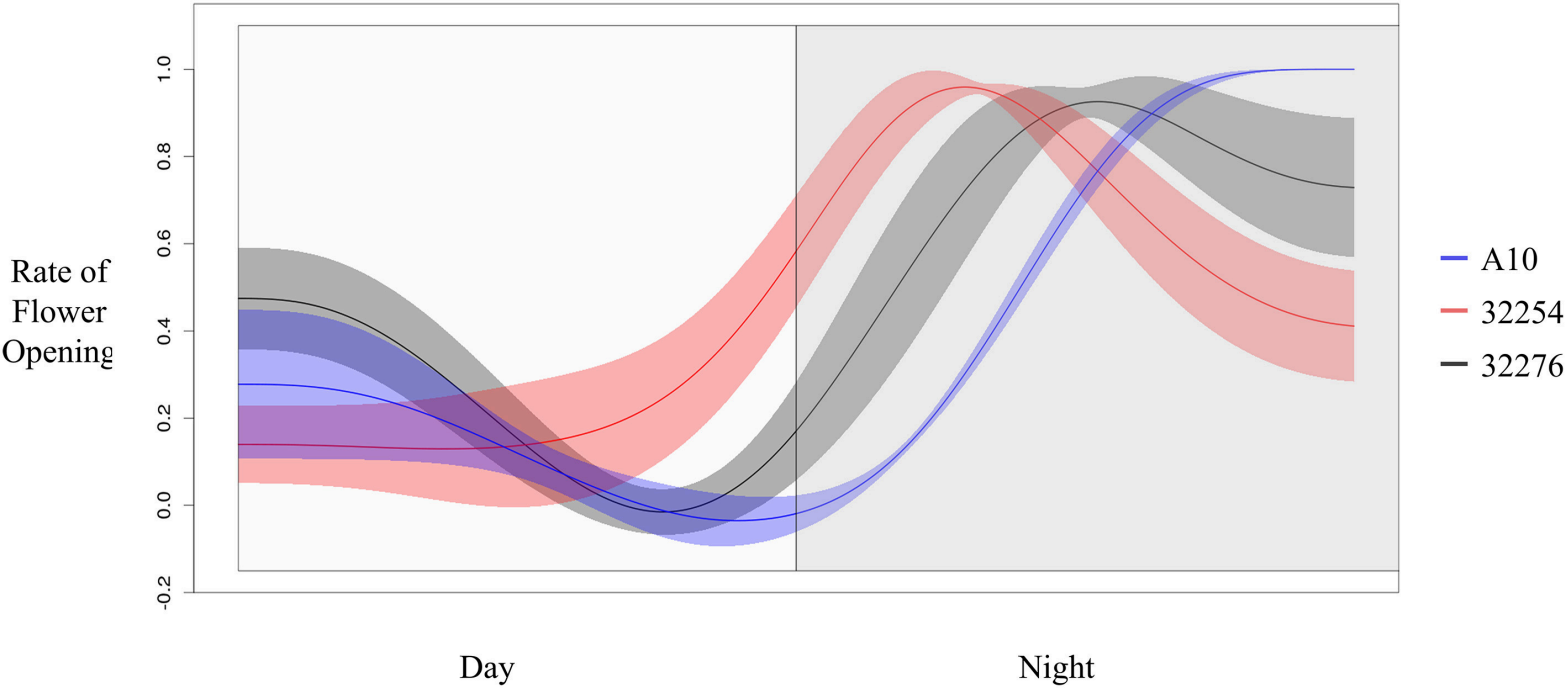
Rate of flower opening of A10 (blue), Ames 32254 (red), and Ames 32276 (black). Rate of flower was normalized by the max rate of each variety. Shading represents the standard error at each timepoint (*N* = 3).

The TAA was determined for three independent panicles for each accession. The rate of flowering was determined by taking the derivative of the ratio of open to closed spikelets over time. We normalized the rate of flower opening by the maximum rate of flowering per panicle. For all three accessions the rate of TAA was highest at night (Figures 4, 5). To ensure that this was not due to differences between the classifiers, we evaluated the calls of the InceptionV3cnn classifier of the first and last image in the light, which confirmed that of the flowers identified as opened during the night. This observation is in agreement with a previous report that flower opening is predominantly restricted to the nighttime hours in *S. viridis* (Rizal et al., 2013). Further evaluation of the specific period in the night when the highest rate of TAA occurs indicates unique temporal differences between the varieties (Figure 5). A10 panicles show a low rate of flowering throughout the first half of the night, and then the flowering rate peaks after midnight and maintains this rate until dawn. Ames 32254 and Ames 32276 show almost no flower opening during the majority of the day period. These accessions appear to initiate flowering before dusk and the peak of flowering rate occurs just after dusk. Flowering rate drops off in Ames 32254 before midnight while the peak rate of flower opening for Ames 32276 occurs in the middle of the night. The variation between accessions indicates that select time intervals can be subset and examined to identify differences in TAA between accessions. For example, the rate of flower opening can be distinguished between the three accessions in the 1.5 h after dusk, but not in the 1.5 h after dawn (Figure 5). An analysis of variance was performed at these two times and showed that the effect of accession type was significant at dusk, but not at dawn [F_Dusk (2,6)_ = 11.68, *p-*value_Dusk_ < 0.01 F_Dawn (2,6)_ = 1.43, *p-*value_Dawn_ > 0.1). *Post hoc* test results of the 1.5 h after dusk time interval indicated that there is a significant difference between all three accessions (Holm's adjusted *p-*value < 0.05).

### Time of leaf emergence varies between the three setaria accessions

**Figure 6:**
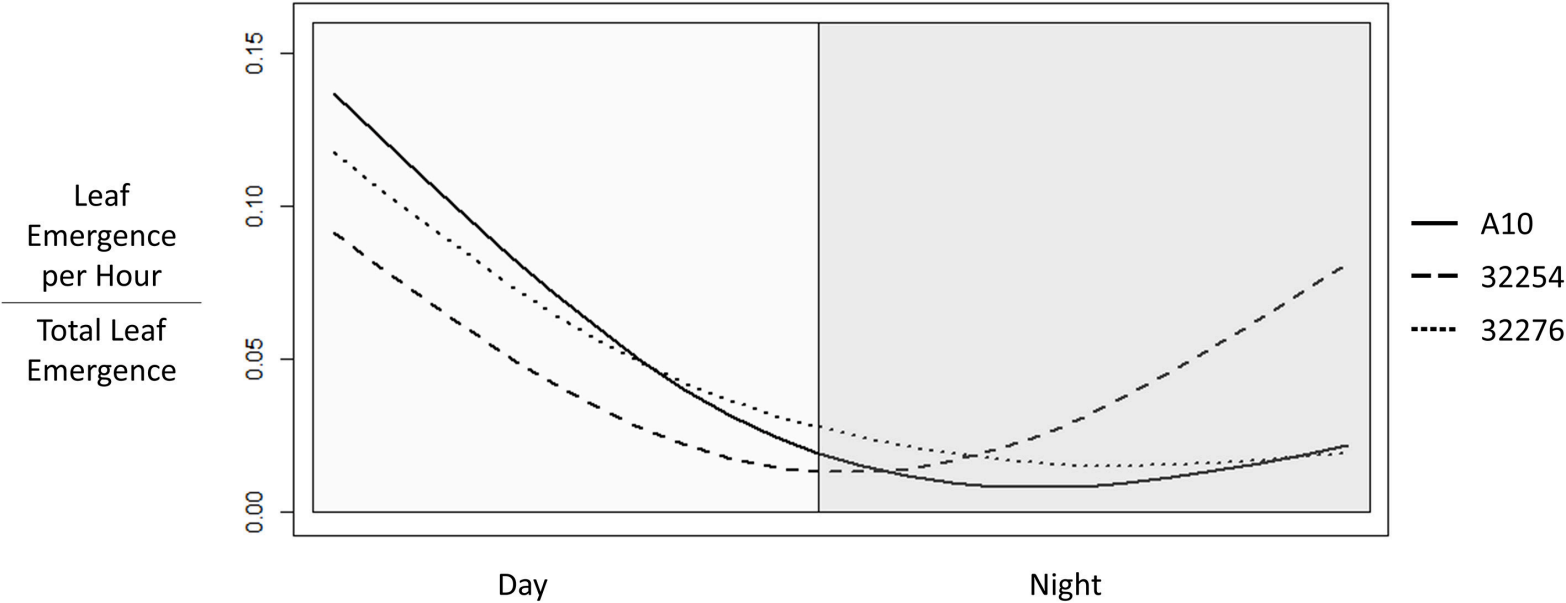
Time of Leaf Emergence events for *Setaria viridis* varieties A10, Ames 32254, and Ames 32276 were determined from hourly images of 3 to 22 day old seedlings. The graph was constructed using the smooth.spline function in R with three degrees of freedom to show the fraction of emergence events at each hour in a 24 h period with day hours being from 8 a.m. to 8 p.m.

Time of leaf emergence has been reported to be under circadian control in Arabidopsis (Kim et al., 2016). To evaluate the potential for variation in time of day of leaf emergence in Setaria between accessions, leaf emergence was evaluated hourly. The time of emergence for accessions A10, Ames 32276 and Ames 32254 were graphed as a fraction of total emergence events observed for each variety (Figure 6). Leaf emergence in accession Ames 32254 begins prior to dawn, with the leaf emergence rate peaking in the period around dawn. In contrast, accessions A10 and Ames 32276 show very low rates of leaf emergence before dawn and the highest rate of leaf emergence in the period just after dawn. Little difference is observed between accessions A10 and Ames 32276. The period prior to dawn provides a clear distinction in leaf emergence rates between accessions A10 and Ames 32254.

### Timing of leaf growth varies between the three setaria accessions

**Figure 7:**
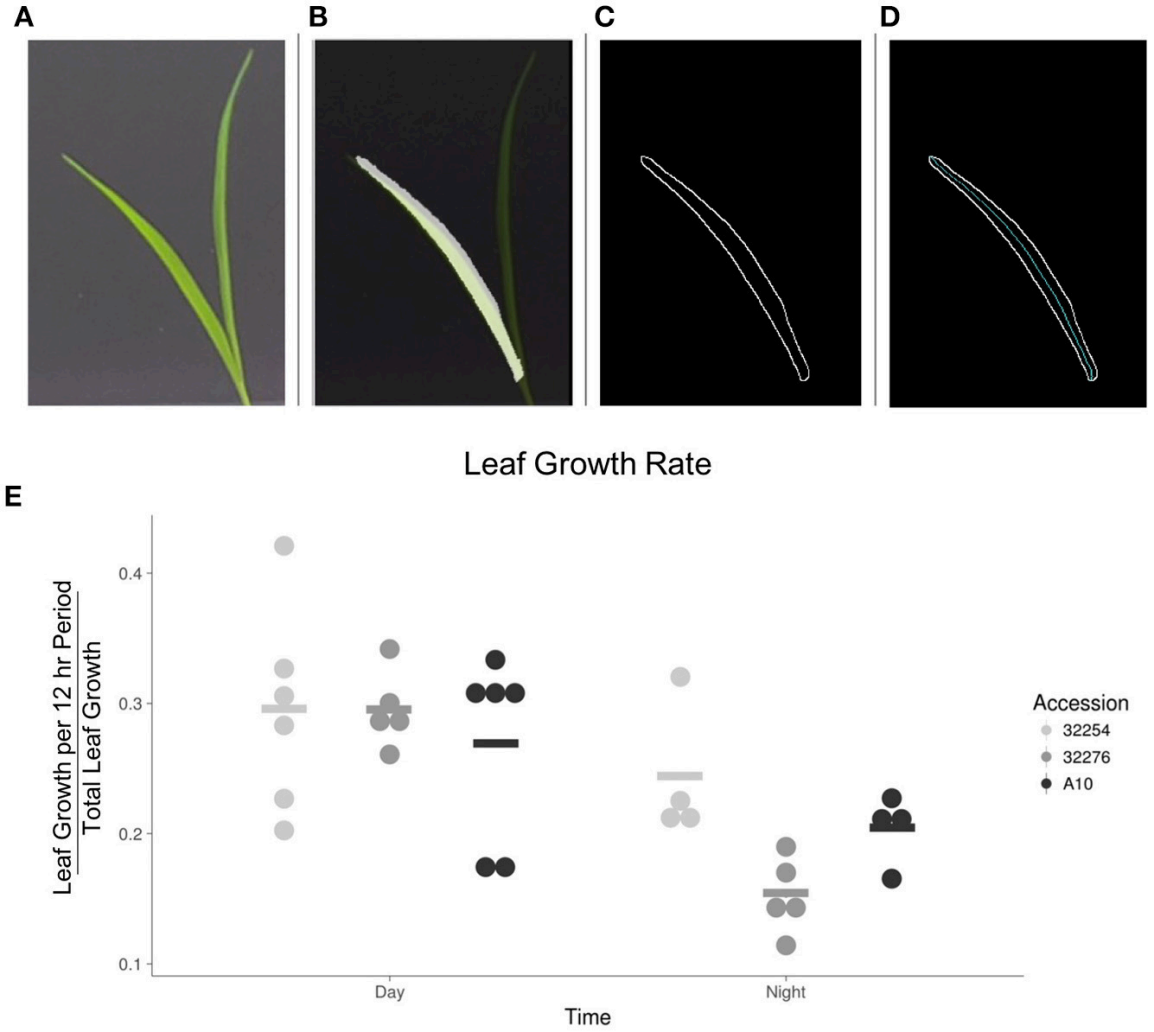
Image analysis of leaf growth. **(A)** Original leaf image (leaf 3, 48 h after emergence). **(B)** Cropped image with a transparent original image overlaid. **(C)** Visualization of edge detection results. **(D)** Visualization of the calculation of the midline used to determine length of leaf. **(E)** Graph of the rate of growth as a percentage of the total growth for the three accessions in the day vs. the night (*N* = 3), bar indicates mean for each accession.

We evaluated leaf growth, broadly distinguishing between how much the leaf grew in the 12 h between dawn and dusk and how much it grew in the night period. The majority of the growth for all three accessions in our conditions occurred in the light period (Figure 7). This is consistent with a previous analysis of *Brachypodium distachyon*, where growth rate was not constrained to a specific time of day, but was primarily responsive to temperature changes (Matos et al., 2014). We observed little variation in the amount of growth between these three accessions during the daytime. A more detailed analysis will be necessary to determine if there is variation in the specific time during the day when growth occurs.

## Discussion

### Advantages of identifying the molecular mechanisms of TAA

Little is known about the genetic regulators of TAA, particularly in monocot species (van Doorn and Kamdee, 2014). Heat escape mechanisms can be used by plants particularly field crops, to avoid high temperature stress by moving heat-sensitive processes, such as TAA and pollination related events to cooler times of the day (Jagadish et al., 2015; Ohama et al., 2017). Pollen development and anthesis have been shown to be highly sensitive to heat stresses in multiple crops including rice, wheat, and sorghum (Satake and Yoshida, 1978; Jagadish et al., 2007; Jain et al., 2007, 2010; Oliver et al., 2007; Ji et al., 2010, 2011). In rice, altering TAA through introgression of genomic regions from earlier flowering wild accessions results in higher spikelet fertility and grain yield under high temperatures (Ishimaru et al., 2012; Hirabayashi et al., 2015; Bheemanahalli et al., 2017). Although the effectiveness of this trait has been demonstrated at the agronomic scale, the molecular components that drive this change are unknown. Yet, without knowing the molecular components driving TAA, the full opportunities for optimizing TAA in rice and other monocot species cannot be achieved. Hence, identifying the molecular mechanisms that alter TAA in monocot species will enable more precise control of TAA in economically important crop species through breeding and/or biotechnology.

Efforts to identify the genetic regulators of TAA could be affected by environmental variation. Characterization of the environmental factors that influence TAA have revealed differences between rice genotypes, which has been reported as impervious to environmental perturbations (Kobayasi et al., 2010) and wheat where TAA is altered by high temperature (Aiqing et al., 2018). Evaluation of TAA in controlled conditions will reduce the potential confounding effects of environmental variables. *Setaria viridis*, with its small stature and rapid generation time (Li and Brutnell, 2011) could serve as a model for TAA in monocot species if genetic variation in TAA exists between Setaria varieties and this could help unravel the relationship between genotype and environment that may influence TAA by controlling environmental conditions in the greenhouse. We developed an image capture and analysis pipeline to classify TAA in *Setaria viridis*, demonstrated that the approach functions well in three different accessions, and identified variation in TAA between the three accessions. With this imaging pipeline, the larger genetic resources available in *Setaria viridis* can be exploited to determine the mechanisms regulating TAA.

### Potential for high-throughput analysis of TAA in setaria

This approach was completed with a single imaging system. Given the low-cost of the Raspberry Pi, NoIR Camera, and IR light system, the throughput of this analysis can be increased. Higher throughput temporal imaging of multiple accessions will face two major challenges: the storage requirements for images and the processing time required for identifying TAA in IR-lit images based on movement. For the daytime images, the InceptionV3cnn approach was able to classify open spikelets in a computationally efficient manner that is suitable for adaptation to high-throughput analysis. The movement-based analysis was also able to classify spikelet opening in daytime images; however, this approach took about ten times longer than the InceptionV3cnn to apply and would not be suitable for large high-throughput datasets. In the IR-images, the InceptionV3cnn approach was not able to distinguish between open and closed spikelets. For these images, the challenge of the amount of time required for processing could be reduced by identifying specific times where differences are greatest between accessions. Additionally, we have not fully explored the effects of image compression or reducing the image quality on the ability to accurately determine TAA. If lower quality images perform as well, this could reduce the storage requirements. Reduced image size may also improve the feasibility of parallelizing the nighttime image analysis. Evaluating options for improving the distribution of individual processes for parallel processing will also enhance the throughput of the nighttime image analysis. Perhaps the most promising approach to improving the throughput is to first screen for differences in the percent of flowers that open in the day vs. night. This evaluation would reduce the required images to two per day and since the first and last image in the light could be used to determine this ratio, the InceptionV3cnn classifier could be used to identify open flowers.

### Additional improvements for image analysis

One challenge in the image analysis pipeline was identifying the spikelets themselves. Our approach, sectioning the panicle image, is appropriate for identifying when the detected flowers open since we only evaluated TAA based on the total spikelets detected. However, this approach may miss individual spikelets and therefore would not be sufficient for quantifying the number of flowers per panicle, size of individual spikelets, and other characteristics of interest for comparing between varieties. This is an area that requires further investigation to identify successful algorithms.

### *Setaria viridis* as a model for TAA

Similar to previously published reports in rice and wheat, in identical growth conditions genetic variation in TAA exists in Setaria. In our growth conditions, the three accessions we evaluated showed the majority of flowers opening at night. Overall TAA was restricted to the nighttime and the period just after dawn. Yet differences in the TAA could be distinguished between the accessions (Figures 4, 5). From 3 to 6 h after dusk, and in the 3 h prior to dawn TAA rates in A10 could be distinguished from Ames 32276 and Ames 32254. The 2 h prior to dusk through 3 h after dusk provided the best time to distinguish between Ames 32276 and Ames 32254. For two Setaria accessions that show variation in TAA, the highest resolution of image acquisition could be concentrated to the time period where there is the greatest difference in the rate of TAA, thus reducing the number of images needed. For some accession comparisons, the total difference in the percent of spikelets that open in the day vs. the night can be distinguished. This difference could be tracked by measuring only the first and last image in the light. For such comparisons, this would greatly reduce the number of images that need to be captured and the analysis could be performed only on daytime images, which are not as computationally expensive for determining TAA.

The environmental plasticity of TAA in Setaria has not yet been examined, but this imaging system can provide the opportunity to determine if Setaria accessions show sensitivity in TAA similar to wheat or are reticent to changes in TAA like rice. This imaging system and platform will enable the evaluation of the effects of temperature, photoperiod, humidity, and stresses on TAA. The role of the circadian clock in regulation of TAA can be evaluated. Furthermore, Setaria cultivars can be examined to evaluate if there is genetic variation in the plasticity of the response to TAA.

We speculate that TAA may be a trait that is under selective pressure during domestication. In rice an evaluation of TAA across multiple wild accessions and domestic cultivars indicated that the domesticated cultivars typically completed TAA within a 1–2 h window (Nishiyama and Blanco, 1981; Prasad et al., 2006; Sheehy et al., 2007; Cattivelli et al., 2008; Bheemanahalli et al., 2017). In contrast, most wild varieties showed a much broader range of TAA (Sheehy et al., 2007). Efforts to reduce outcrossing and genetic variation in domesticated cultivars may inadvertently select for a narrow TAA. Alternatively, a narrow range of TAA may be linked to beneficial yield traits. It is important to note that the A10 accession has been lab-grown for several generations, while the Ames 32276 and Ames 32254 accessions were more recently collected. The differences we observe here could be due to the selection of A10 under growth conditions distinct from the other accessions, perhaps indicating an early stage of domestication. Alternatively, there could be epigenetic differences in A10 due to the continued growth in laboratory conditions. The Setaria model system provides the opportunity to examine the sources of variation in TAA between wild *Setaria viridis* accessions and domesticated *Setaria italica* cultivars (Bennetzen et al., 2012; Mauro-Herrera et al., 2013; Qie et al., 2014).

## Author contributions

ES, BG, SVKJ, OW, and CD conceived of the original concept. ES optimized Setaria growth and cultivation conditions to acquire healthy panicles for imaging. ES and JD optimized camera imaging. JD developed the script for optimizing camera parameters. JD identified, tested, and optimized the image processing and analysis algorithms for detecting TAA. JD and ES performed manual validation of TAA calls. JD and DL optimized image acquisition of setaria seedlings. JF developed the analysis of bristle density, leaf growth, and panicle growth. JF and DL optimized image processing and analysis of seedling growth. JD, ES, DL, JF, BG, SVKJ, OW, and CD contributed to the manuscript preparation including figure production, writing, and editing.

### Conflict of interest statement

BG was employed by Benson Hill Biosystems. The remaining authors declare that the research was conducted in the absence of any commercial or financial relationships that could be construed as a potential conflict of interest.
