# Supplementary material for: Neural Net Classification Combined With Movement Analysis to Evaluate Setaria viridis as a Model System for Time of Day of Anther Appearance
Source: Front Plant Sci. 2018 Oct 31;9:1585. doi: 10.3389/fpls.2018.01585 (PMC6220418; doi:10.3389/fpls.2018.01585)
Supplement: Supplementary file 7 [file Presentation_1.PDF]

Supplementary Material

Neural Net Classification Combined with Movement Analysis to Evaluate *Setaria viridis* as a Model System for Time of Day of Anther Appearance

Jigar S. Desai, Erin Slabaugh, Donna J. Liebelt, Jacob D. Fredenberg, Benjamin N. Gray, SV Krishna Jagadish, Olivia Wilkins, Colleen J. Doherty*

*** Correspondence:** Colleen J. Doherty: colleen_doherty@ncsu.edu

1 Supplementary Data

Video 1. True positive example of correct classification of TAA in nighttime images. Night time images of spikelet classified as opening, that does open at time identified.

Video 2. : True negative example of correct classification of TAA in nighttime images. Night time images of a spikelet classified as non-flowering in this image series that does not open during this time period.

Video 3. False negative example of incorrect classification of TAA in nighttime images. Night time images of a spikelet classified as non-opening during the imaging period that does open during this period.

Video 4. Nighttime images of an A10 panicle.

Video 5. Nighttime images of an Ames 32254 panicle.

Video 6. Nighttime images of an Ames 32276 panicle.

2 Supplementary Figures and Tables

2.1 Supplementary Figures

Variety	Latitude	Collected from:
A10 (PI669942)	--	Manitoba, Canada
32254	49.64306000	Manitoba, Canada
32276	44.82616900	Bordeaux, France

Supplementary Figure 1. The *Setaria viridis* accessions evaluated and their originating location. While A10 originated in Manitoba, Canada, it has been used in the laboratory for many generations and the seeds we evaluated were from lab-propagated sources. (All information in this table was obtained from GRIN accession archives).

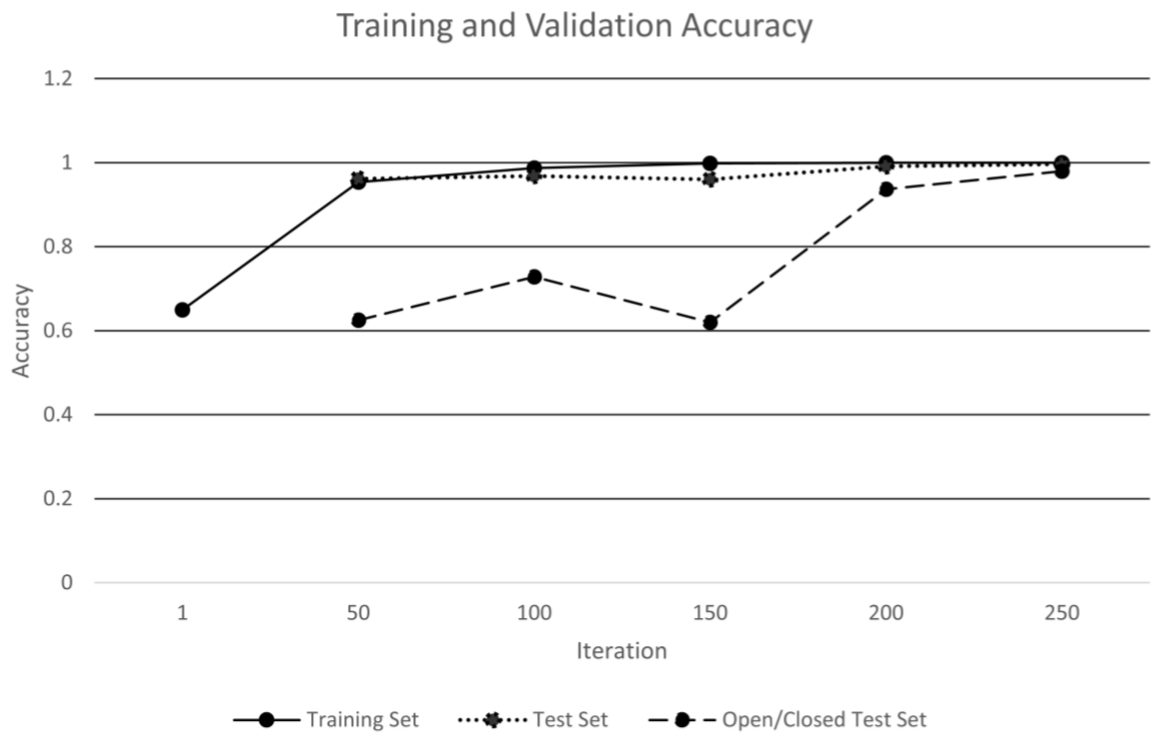

Supplementary Figure 2. The performance of the InceptionV3cnn model per iteration. The solid line indicates the accuracy on the training data set, which was unbalanced and included background, open, and closed spikelets. The dotted line indicates the performance on the entire test set which contained background images and an equal number of open and closed spikelets. The dashed line shows the accuracy when evaluating the performance on the criteria of interest, validation of open versus closed spikelets, a balanced test set.

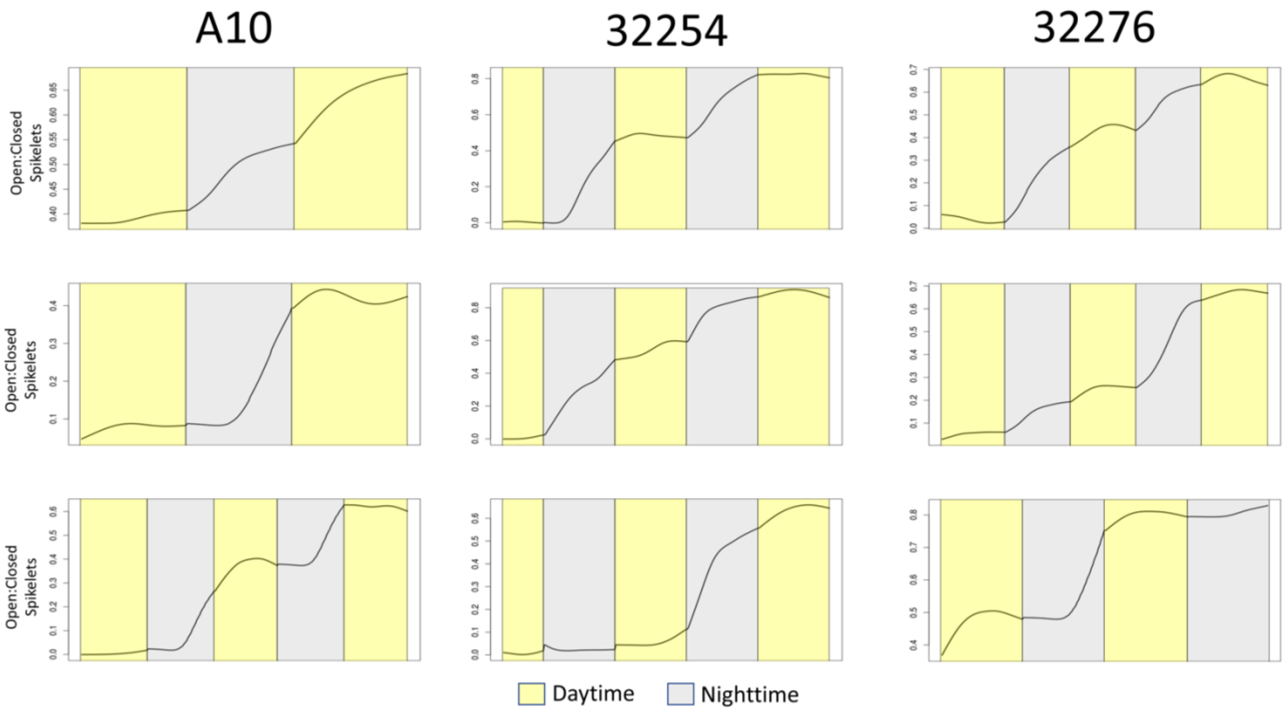

Supplementary Figure 3. Ratio of open to closed spikelets for A10, 32254, and 32276. Each setaria accession had three biological replicates represent by the three plots for each accession. Time of flowering was observed from 1.5 to 2.5 days. Day (yellow) and night (grey) time points were combined for each accession to find the rate of flowering.
